# Participation with alcohol marketing and user-created promotion on social media, and the association with higher-risk alcohol consumption and brand identification among adolescents in the UK

**DOI:** 10.1080/16066359.2019.1567715

**Published:** 2019-02-19

**Authors:** Nathan Critchlow, Anne Marie MacKintosh, Lucie Hooper, Christopher Thomas, Jyotsna Vohra

**Affiliations:** a Institute for Social Marketing and UK Centre for Tobacco and Alcohol Studies, Faculty of Health Sciences and Sport, University of Stirling, Stirling, Scotland;; b Cancer Policy Research Centre, Cancer Research UK, London, UK

**Keywords:** Alcohol marketing, social media, brand identification, young people, alcohol consumption

## Abstract

**Aim:** To explore participation with alcohol marketing (i.e. commenting on brand statuses) and user-created promotion on social media (i.e. photos of peers drinking) by young people in the United Kingdom (UK), and what association this has with higher-risk consumption and brand identification.

**Method:** Online cross-sectional survey with 11–19-year olds in the UK (*n* = 3,399) (average age: 15 years old). Past-month participation was measured for five forms of alcohol marketing on social media and one form of user-created promotion (all Yes/No). Past-month awareness of nine wider alcohol marketing activities, social media apps used at least weekly, and ownership of branded merchandise were included as covariates. Outcomes included higher-risk consumption in current drinkers (≥5 AUDIT-C) and brand identification in all respondents (8 pictures with brand names removed).

**Results:** Over one-in-ten respondents (13.2%) had participated with at least one form of marketing on social media or participated with user-created promotion (12.2%). For both, participation was greater in current drinkers and those of legal purchasing age. A logistic regression found that participation with two or more forms of marketing on social media (*AOR* = 1.96, *p* < .01) and participation with user-created promotion (*AOR* = 3.46, *p* < .001) were associated with higher-risk drinking. Respondents, on average, identified 2.58 (*SD* = 2.12) alcohol brands. A linear regression found participation with marketing on social media was not associated with brand identification (*β* = 0.01, *p* = .42) but participation with user-created promotion was (*β* = 0.05, *p* < .001).

**Conclusion:** Social media provides opportunities for adolescents to participate with commercial marketing and user-created promotion and this is associated with higher-risk consumption and brand identification.

## Introduction

The relationship between alcohol marketing and consumption in adolescents and young adults (young people) has been a topic of debate for decades (Gordon et al. 2009), with systematic reviews reporting a causal relationship between exposure and consumption (Anderson et al. 2009; Jernigan et al. 2017). Branding is also an important part of the alcohol marketing process (Hastings et al. 2010; Roberts et al. 2016), with research finding that young people are knowledgeable of brand names, associate brands with positive and desirable identities, and that owning branded merchandise or having a favourite brand is associated with consumption (McClure et al. 2013; Morey et al. 2017; Purves et al. 2018). Alcohol marketing has embraced social media to find new ways to influence and interact with consumers (Carah 2017; Lobstein et al. 2017) and research supports that such online marketing does influence health behaviours (Dunlop et al. 2016; Gupta et al. 2016; Lobstein et al. 2017; Buchanan et al. 2018). Young people in the UK report extensive use of social media (OFCOM 2017a, 2017b). It is therefore important to explore how this may facilitate exposure to content which promotes alcohol and what association (if any) this has with consumption and marketing goals.

Online is the fastest growing advertising medium in the UK, and expenditure (£10.3 billion) is double that of television advertising (£5.2 billion) (OFCOM 2017c). The advertising revenue received by website operators further highlights that developing innovative marketing is highly profitable, with Facebook ($29.6 billion) and Google ($79.4 billion) receiving one-fifth of global advertising spend (Kollewe 2017). New media technology has created a ‘digital marketing mix’ which includes ‘paid for’ advertising (e.g. pop-ups), ‘owned media’ (e.g. websites), and ‘earned media’ (e.g. user-generated content on social media) (Arnhold 2010). Compared to traditional media (e.g. television), social media has several commercial advantages, including the ability to target marketing at specific audiences, virally spread content, extend the reach of traditional media, allow marketing to be accessed in almost any context, and actively engage users in the marketing process (Chaffey and Ellis-Chadwick 2012).

Research which has explored alcohol marketing through digital and social media can be grouped into two categories. The first, content research, reports that digital marketing features prominently online (from social media pages to smartphone apps), has a global reach, is updated continuously, and plays an important role in ‘360-degree’ marketing strategies (Mart et al. 2009; Chester et al. 2010; Nicholls 2012; Weaver et al. 2013; Barry et al. 2015; Barry et al. 2016; Carah et al. 2018). Content research also suggests that alcohol marketing through social media uses a variety of creative strategies to appeal and invests significant resources into creating real-time and topical connections which reflect the brand identity and instigate interactions with consumers (Brooks 2010; Nicholls 2012; Atkinson et al. 2014; Carah et al. 2015). Content research has also found that age-verification measures are often underpinned by weak designs and have limited efficacy for preventing access by underage profiles (Jones et al. 2014; Barry et al. 2015; Barry et al. 2016), and that content may appeal to younger audiences directly through creative strategies (e.g. ‘advergaming’) or indirectly by tying the brand to topical and cultural associations which resonate with younger audiences (Griffiths and Casswell, 2010; Atkinson et al. 2014; Carah et al. 2015; Purves et al. 2014). Content research has also raised concerns that alcohol marketing on social media does not always adhere to regulations and may contain minimal or ambiguous promotion of lower-risk consumption (Brooks 2010; Nicholls 2012; Carah et al. 2015).

The second category, consumer research, explores how digital marketing influences attitudes and consumption. Self-report surveys find that young people are aware of, and participating with, a range of alcohol marketing on digital and social media, and that this is associated with increased consumption, heavy-episodic drinking, and positive expectancies (de Bruijn et al. 2012; Critchlow et al. 2016; McClure et al. 2016; Jernigan et al. 2017). Survey research also reports that the association between digital marketing and consumption is stronger when the audience participates (e.g. commenting on a brand status) and that participation with digital marketing has stronger association with consumption than traditional marketing (Gordon et al. 2011; Critchlow et al. 2016). Experimental evidence also suggests that the effect of alcohol marketing on social media is amplified when content appears to have been positively received by other users and that marketing may even be more powerful than messages about lower-risk drinking presented at the same time (Alhabash et al. 2015; Alhabash et al. 2016). Qualitative research further reports that young people are knowledgeable of alcohol marketing on social media, consider it to portray consumption in a positive manner, view it as a normal and ubiquitous part of online experiences, consider branding to hold cultural and symbolic value which facilitates identify construction and peer socialisation, and are motivated to participate to receive rewards (e.g. competitions) or for social pleasure (Atkinson et al. 2014; Lyons et al. 2014; Moraes et al. 2014; Weaver et al. 2016; Purves et al. 2018).

In addition to commercial marketing, there are two ways that user content on social media can promote consumption. The first is through user-generated branding, for example sharing a brand status or co-creating content (e.g. fan photos or comments). User-generated branding is significant because it extends the reach of marketing (Arnhold 2010), blurs the boundaries between commercial and peer activity (Lyons et al. 2014), enhances the credibility of the marketing message through peer endorsement (Atkinson et al. 2017), and because such content often falls beyond the brand-controlled spaces defined in regulation (Portman Group 2009). The second method is through user-created promotion, defined as online content which promotes consumption independent of commercial influence or involvement (Critchlow et al. 2017). Although this definition covers a variety of content, ranging from online videos (Primack et al. 2015) to smartphone apps (Weaver et al. 2013), status updates or photos of the self or peers drinking posted on social media are frequently cited (Moreno et al. 2016; Critchlow et al. 2017). Similar to commercial-led marketing, research has found that young people are aware of user-created promotion on social media, are willing to both participate with existing content and create their own, and that doing so is associated with increased consumption (Boyle et al. 2016; Thompson and Romo 2016; Critchlow et al. 2017). That user-created promotion also frequently features alcoholic products and brand iconography, thus providing free brand exposure (Primack et al. 2015), also helps to explain how such content may contribute to wider marketing goals of raising brand awareness and influencing consumption.

In the UK, there is a growing research exploring participation with marketing and user-created promotion on social media and the possible links to consumption (Atkinson et al. 2014; Critchlow et al. 2016, 2017; Purves et al. 2018). To date, however, the single-item measures used to assess adolescent participation with online marketing underestimate the varied ways in which audiences can engage. For user-created promotion, research in the UK has mostly focussed on young adults above the legal drinking age (Moss et al. 2015; Critchlow et al. 2017). Further research is needed to understand participation by adolescents and what role (if any) it may play in shaping consumption. Furthermore, no research in the UK has measured participation with both commercial marketing and user-created promotion simultaneously, and the combined association with they have with consumption. Finally, research has mostly focussed on consumption as an outcome, and does not consider how social media contributes to wider marketing goals, for example being associated brand identification.

In this study we explore participation with alcohol marketing and user-created promotion on social media. We also explore the association that this has with higher-risk consumption and brand identification in young people in the UK, after controlling for relevant demographic factors and co-variates associated with consumption. We did not adopt *a priori* hypotheses.

## Methods

### Design and sample

Data were collected through the 2017 Youth Alcohol Policy Survey, an online cross-sectional survey conducted with 11–19-year olds in the UK (*n* = 3,399). Responses were collected April–May 2017. The survey was hosted by YouGov, a market research company, who recruited a representative sample from their existing UK panel (YouGov 2017). Participants aged ≥16 years old were approached directly to participate (and received 150 points, equivalent to £1.50, on their YouGov account). Respondents under 16 years old were approached through existing adult panel members (and received 250 points, equivalent to £2.50, on their YouGov account). Age (11–19 years old) and membership of the YouGov panel (directly or indirectly through an adult) were the only inclusion criteria. A survey weight (based on age, gender, ethnicity, region and social grade) was provided to allow descriptive data to be representative of the UK population.

### Measures

#### Demography

Age, gender, ethnicity, resident country (England, Scotland, Wales, Northern Ireland), living status, employment status, educational status, legal purchasing status (Yes/No), and indices of multiple deprivation (IMD) – a measure of deprivation within local areas based seven elements such as income, crime, and education (Department for Communities and Local Government 2015) – were obtained from information held about respondents or survey questions. Details of the demographic categories are reported in Table One.

#### Social media apps used at least weekly

Participants were prompted with the phrase ‘*Which, if any, of the following apps do you use at least once a week?’* and presented with a list of 10 apps: (1) Facebook; (2) Instagram; (3) Pinterest; (4) Snapchat; (5) Spotify; (6) Tumblr; (7) Twitter; (8) WhatsApp; (9) YouTube; and (10) Other, with free text box to write in. Participants were asked to tick all which applied (Yes/No). Participants were also presented the option ‘None of the above’. A cumulative score was computed for social media apps used at least weekly (0–10), and respondents were split into tertiles of high (six or more apps), medium (four or five), and low use (three or fewer).

#### Participation with alcohol marketing on social media

Participation was measured for five forms of commercial marketing on social media: (1) Liked an alcohol brand on social media, such as Twitter, Facebook or Instagram; (2) Shared something related to an alcohol drinks brand, such as a status, Tweet, or picture; (3) Followed an alcohol brand on social media; (4) Entered a competition run by an alcoholic drinks brand online or on social media; and (5) Searched for alcoholic drinks adverts on websites, such as YouTube. Participants were prompted with the statement ‘*Which, if any, of the following have you done in the last month?’* and asked to self-report those they had participated with (Yes/No). Participants could also indicate ‘none of the above’. A cumulative score was computed (0–5). Respondents were also classified into those who had not participated with any marketing, those who had participated with one form, and those who had participated with two or more.

#### Participation with user-created alcohol promotion

Participants were asked to indicate if they had updated their status or uploaded pictures of themselves or friends drinking an alcoholic drink in the past month (Yes/No).

#### Alcohol consumption and higher-risk drinking

Drinking status was measured through the item ‘*Have you ever had a whole alcoholic drink? Not just a sip.*’ (Yes/No/Prefer not to say). Those who answered ‘Yes’ were classified as ‘ever-drinkers’ and those who answered ‘No’ classed as ‘never-drinkers’.

Consumption in ‘ever-drinkers’ was measured through the Alcohol Use Disorders Identification Test – Consumption (AUDIT-C). The scale assessed three behaviours: (1) frequency of consumption; (2) units drunk in a typical drinking occasion; and (3) frequency of heavy episodic drinking. Responses were provided on scales scored 0–4, with the answers relative to frequency (0 = Never – 4 = Four or more times a week), units drunk (0 = 1–2 units – 4 = 10 or more units), and frequency of heavy-episodic drinking (0 = Never – 4 = Daily or Almost Daily). An AUDIT-C score was computed (0–12), and a cut off of ≥5 was used to identify higher-risk consumption (Research in Practice 2015; [PHE] Public Health England 2017). Participants who answered other than ‘never’ on the first AUDIT-C item were categorised as ‘current drinkers’ and those who answered ‘never’ were categorised as ‘non-drinkers’.

#### Brand identification

To measure brand identification, participants were shown eight visuals of alcohol brands with the name removed and asked to write in the correct name (Harris et al. 2015). The stimuli included spirit, wine, cider, beer and alcopops/ready-to-drink brands, chosen to represent a variety of popular product types and likely market appeal (brands are reported in the results). For each brand, the written answer was coded as correct or incorrect/blank. Misspellings or well-known abbreviations of brand names (e.g. ‘Jager’ not ‘Jägermeister’) were coded correct (Henriksen et al. 2008; Harris et al. 2015). A cumulative score was computed for identification (0–8).

#### Awareness of wider alcohol marketing activity and ownership of branded merchandise

Social media is only one component of the alcohol marketing mix (AFS 2017). To account for this, and contextualise any association between social media marketing and consumption, a measure was taken for awareness of nine wider forms of alcohol marketing activity in the past month (Supplementary Table S1). For each alcohol marketing activity, the self-reported frequency of awareness (1 = Everyday – 6 = Not in the past month; Not sure) was converted into the estimated number of days that alcohol marketing was seen across a four-week period (‘one month’) (e.g. seen ‘Everyday’=seen 28 times in four weeks). A cumulative score for monthly awareness was created across all nine alcohol marketing activities and the sample were separated into tertiles of low awareness (≤16 instances per month), medium (17–53 instances), high (≥54 instances), and a fourth category for participants who indicated ‘not sure’ to any of the nine examples of marketing.

Participants were also asked to indicate whether they owned any alcohol branded merchandise (Yes/No/Not sure).

#### Covariates

Several covariates were included to contextualise any association between marketing, user-created promotion, and alcohol consumption. Covariates were chosen to mirror those included in previous research into digital marketing (Gordon et al. 2011; Lin et al. 2012) and interpersonal factors associated with consumption (Schelleman-Offermans 2012; Leung et al. 2014). Frequency of consumption was measured for the mother (female carer), father (male carer), and closest friend (each scored: 1 = Never – 9 = Every day or almost every day; Prefer not to say; Not applicable). For each, the frequency was converted into five categories: Never, Less than monthly, Monthly or fortnightly, At least weekly, and Not stated. Perceived acceptability of drinking an alcoholic drink (a whole drink, not just a sip) was measured for parents and peers (each scored: 1 = Total acceptable – 5 = Totally unacceptable). For both parents and peers, acceptability was converted into three categories: Neutral or unacceptable; Acceptable; and Not stated. For current drinkers, age of first drink was also measured (<8 years–19 years; Can’t remember or Prefer not to say). Answers were converted into four categories (age ≤13; age 14–15; age ≥16; and not stated).

#### Ethics

Ethical approval was obtained from the University of Stirling’s General University Ethics Panel (GUEP59). YouGov also included a lead for ethical and quality assurance, including informed consent, post-survey debriefing and signposting to support organisations, and confidentiality and anonymity of responses.

#### Analysis

Data were analysed in SPSS version 23. Descriptive data were weighted so percentages and mean scores were representative of the demographic profile of the UK population. Bivariate analyses, based on Chi-square tests and one-way Analyses of Variance (ANOVA, or Welch *F* where homogeneity of variance was not assumed), examined how participation with alcohol marketing on social media, participation with user-created promotion, and level of brand identification, differed by current drinking and legal-purchasing age status.

Multivariate analyses were conducted on unweighted data as the demographic and confounding variables were controlled for in the regressions. A hierarchical logistic regression was conducted with higher-risk drinking (≥5 AUDIT-C) in current drinkers as the dependent variable. A hierarchical linear regression was conducted on all participants with brand identification as the dependent variable (0–8). The key independent variables in both models were participation with alcohol marketing on social media, participation with user-created promotion, and number of social media apps used at least weekly. Models controlled for age; gender; ethnicity; IMD quintile; resident country (Northern Ireland and Wales were combined due to small sample sizes); educational status; employment status; frequency of mother (female carer), father (male carer) and close peer consumption; perceived parental and peer acceptability of consumption; age of first drink; awareness of wider alcohol marketing activity; and ownership of branded merchandise. Country, ethnicity and educational status were excluded from the logistic regression to avoid over-saturation and because they were non-significant in earlier stages of the analysis. As the linear regression was based on the entire sample, it additionally controlled for consumption (non-current drinker, lower-risk drinker, and higher-risk drinker). In the logistic regression, where the categorical variables had three ≥3 levels, and were of an ordinal level, the SPSS contrast = difference function enabled comparison of each increasing category relative to the combined previous categories. In the linear regression, categorical variables with ≥3 categories were converted into dummy (binary) variables to aid comparison. The omitted dummy variable formed the reference category. For example, age of first drink had four levels and three binary variables were computed: first drink aged 13 or under, first drink aged 14–15 years old, first drink aged 16 years or older, and not stated. By including 13 years or under, 16 years or older, and not stated in the model, having first drink aged 14–15 years old was the reference category.

## Results

### Sample profile and alcohol use

The weighted sample (*n* = 3,399) had an average age of 15.18 years old (*SD* = 2.55), had an even proportion of males and females, and even distribution across IMD quintiles (Table 1). After excluding cases with missing data on drinking status (*n* = 60), almost half of the sample (48%, *n* = 1,590, weighted) were current drinkers and almost half of current drinkers (44%, *n* = 707, weighted) were classed as higher-risk.

**Table 1.** Sample profile based on unweighted and weighted frequencies.

	Unweighted		Weighted	
Variable	*n*	%	*n*	%
Gender				
Male	1,679	49	1,733	51
Female	1,720	51	1,666	49
Legal purchase age for alcohol				
No	2,551	75	2,582	76
Yes	848	25	817	24
Ethnicity				
White British	2,716	80	2,594	76
Other	647	19	779	23
Not specified	36	1	26	1
Country lived in				
England	2,601	77	2,869	84
Scotland	424	12	265	8
Wales	250	7	160	5
Northern Ireland	124	4	105	3
IMD Quintile^a^				
1 (most deprived)	680	20	676	20
2	666	20	676	20
3	723	21	676	20
4	616	18	676	20
5 (least deprived)	712	21	676	20
Living arrangements				
At home with parents or adult family member	3,044	90	3,048	90
Other	355	10	351	10
In education^b^	3,205	95	3,216	95
In employment^c^	254	8	244	7
Drinking status^d^				
Current drinkers	1,615	48	1,590	48
Non-drinker	1,724	52	1747	52
Higher-risk consumption				
Yes	708	21	707	21
No	2,631	56	2,630	56

^a^Cases excluded due to missing data =2; ^b^=14; ^c^=14; ^d^=60.

### Social media apps used at least weekly

Almost all respondents used at least one social media app at least weekly (95%). Respondents, on average, used 3.89 of the 10 social media apps at least weekly (*SD* = 2.10). Twenty five per cent of the sample were considered high social media app users, 33% were considered medium, and 42% were considered low.

### Participation with alcohol marketing on social media

More than one-in-ten respondents (13.2%) had participated with at least one form of alcohol marketing on social media (Table 2). Around one-in-twenty had liked an alcohol brand on social media (5.5%) or shared an alcohol brand status, tweet or picture (5.9%). Fewer than 5% had searched for drinks adverts, followed an alcohol brand, or entered a competition. Participation with a least one form of marketing was higher in current-drinkers (22.4%) compared to non-drinkers (5.0%), χ^2^(1)=219.15, *p* < .001. It was also higher for those above the legal purchase age (22.6%) compared to those under (10.3%), χ^2^(1) = 82.82, *p* < .001.

**Table 2.** Participation with alcohol marketing and user-created promotion on social media young people in the UK, by current drinking status.

	Overall (*n* = 3,399)		Current drinkers (*n* = 1,590)		Non-Drinkers (*n* = 1,747)		*Significance*	
	*n*	%	*n*	%	*n*	%	χ^2^	*p*
Digital marketing channel								
Liked alcohol brand on social media	188	5.5	156	9.8	29	1.7	105.62	<.001
Shared alcohol brand status, tweet, or picture	201	5.9	177	11.1	23	1.3	142.34	<.001
Searched for drinks advert (e.g. on YouTube)	131	3.9	99	6.2	30	1.7	45.54	<.001
Followed brand on social media	115	3.4	95	6.0	19	1.1	60.26	<.001
Entered competition on social media	100	2.9	75	4.7	23	1.3	33.80	<.001
Participated with at least one of the above forms of marketing	450	13.2	356	22.4	87	5.0	219.15	<.001
								
User-created alcohol promotion								
Uploading status or pictures of self or friends drinking	415	12.2	390	24.5	19	1.1	425.05	<.001

### Participation with user-created promotion on social media

Over one-in-ten young people (12.2%) had participated with user-created promotion by uploading statuses or pictures of themselves or friends drinking an alcoholic drink (Table 2). Doing so was higher in current drinkers (24.5%) compared to non-current drinkers (1.1%), χ^2^(1) = 425.05, *p* < .001. It was also higher in those above the legal purchasing age (26.2%) compared to those below (7.8%), χ^2^(1) = 196.21, *p* < .001.

### Awareness of wider alcohol marketing activity and ownership of branded merchandise

Of the participants for whom an awareness score could be computed for wider alcohol marketing (i.e. those who had not indicated ‘not sure’ to any of the nine marketing activities; 41%, *n* = 1,401), 35% were classified as having low awareness (≤16 instances per month), 32% had medium awareness (17–53 instances), and 34% had high awareness (≥54 instances). The remaining participants were classed as ‘not sure’. Seventeen per cent owned branded merchandise.

### Brand identification

Respondents, on average, correctly identified 2.58 of the eight alcohol brands (*SD* = 2.12). Three quarters correctly identified at least one brand (76%). Of the eight brands, identification was highest for Foster’s (56%), Malibu (49%), and WKD (49%) (Table 3). One-way ANOVAs indicated that, on average, identification was greater in current drinkers (*M* = 3.91, *SD* = 1.84) compared to non-drinkers (*M* = 1.37, *SD* = 1.55), Welch’s *F* (1,3117)=1,832.19, *p* < .001, *d* = 1.49, and in those above the legal purchasing age (*M* = 4.13, *SD* = 1.94) compared to those below (*M* = 2.08, *SD* = 1.92), *F* (1, 3397)=697.43, *p <* .001, *d* = 1.06.

**Table 3.** Identification of alcohol brands, by current drinking status.

	Overall (*n* = 3,399)		Current drinkers(*n* = 1,590)		Non-drinkers(*n* = 1,747)		*Significance*	
Alcohol brand	*n*	%	*n*	%	*n*	%	χ^2^	*p*
Fosters	1915	56.3	1191	74.9	695	39.8	417.85	<.001
Malibu Rum	1667	49.0	1153	72.5	485	27.8	667.11	<.001
WKD	1650	48.6	1156	72.8	464	26.6	710.74	<.001
Smirnoff	1546	45.5	1128	70.9	392	22.4	789.62	<.001
Jägermeister	1041	30.6	846	53.2	183	10.5	712.71	<.001
Kopparberg	561	16.5	425	26.7	129	7.4	225.26	<.001
Echo Falls	285	8.4	247	15.5	34	1.9	199.47	<.001
Hardy’s	88	2.6	70	4.4	17	1.0	38.59	<.001
Average (SD)	2.58 (2.12)		3.91 (1.84)		1.37 (1.55)		1,832.19^a^	<.001

^a^Between group comparison based on Welch *F*.

### Association between participation with marketing and user-created promotion on social media and higher-risk consumption

A logistic regression, controlling for demographics and covariates, indicated that being a higher-risk drinker was positively associated with participation with marketing on social media (*p* < .05) (Table 4). Current drinkers who had engaged with two or more forms of marketing on social media were almost twice as likely to be higher-risk drinkers vs. those who did not participate with any (*AOR* = 1.96, *p* < .01). In addition, those who participated with user-created promotion were more than three times as likely to be higher-risk drinkers, vs. those who did not (*AOR* = 3.46, *p* < .001). Other marketing and digital variables associated with higher-risk drinking were at least medium awareness of wider alcohol marketing activity (*AOR = 1.82*, *p* < .05) (vs. low awareness); ownership of branded merchandise (*AOR* = 1.45, *p* < .01); and use of a greater number of social media apps at least weekly (e.g. *AOR* = 1.59, *p* < .01, for those using six or more social media apps vs. three or fewer). Drinking behaviours were also significant factors. Having close friends who drink at least weekly (*AOR* = 2.97, *p* < .001) was associated with higher risk drinking, while those having a first drink at aged 16 or older (*AOR* = 0.27, *p* < .001) were less likely to be higher-risk drinkers (vs. those who had their first drink under age 16). Likelihood of higher-risk drinking was also lower for those whose mother drank less than monthly (*AOR* = 0.43, *p* < .01) vs. those whose mother never drank alcohol. Demographic variables associated with being a higher-risk drinker included: being older (*AOR* = 1.33, *p* < .001); male (*AOR* = 1.67, *p* < .001); from a more affluent quintile of deprivation (*AOR* = 1.76, *p <* .05, for the second most deprived quintile vs. the most deprived); and living independent of parents or adult family members (*AOR* = 1.68, *p* < .01).

**Table 4.** Association between participation with alcohol marketing and user-created promotion on social media and higher-risk consumption in current drinkers.

	*n*	AOR^a^	95% CI Lower	95% CI Upper	*p*
Age	1,591	1.33	1.21	1.46	<.001
Gender					
Female	821	Ref			
Male	770	1.67	1.29	2.16	<.001
IMD Quintile					.035
1 (most deprived)	228	Ref			
2 *v 1*	336	1.76	1.13	2.74	.012
3 *v 1 & 2*	325	1.37	0.97	1.95	.077
4 *v 1–3*	346	1.26	0.93	1.72	.143
5 (most affluent) *v 1–4*	356	1.27	0.95	1.70	.112
Working status					
Not working	1,378	Ref			
Working (full or part-time)	213	1.13	0.79	1.63	.504
Living status					
Living with parents or adult family	1,320	Ref			
Living independently	271	1.68	1.16	2.42	.006
Frequency of mother drinking					.011
Never	115	Ref			
Less than monthly *v never*	282	0.43	0.25	0.76	.003
Monthly or fortnightly *v less often*	281	1.12	0.75	1.67	.589
At least weekly *v less often*	849	0.92	0.68	1.23	.561
Not stated *v all other categories*	64	1.68	0.85	3.29	.133
Frequency of father drinking					.236
Never	77	Ref			
Less than monthly *v never*	159	1.16	0.59	2.32	.664
Monthly or fortnightly *v less often*	202	0.68	0.41	1.11	.126
At least weekly *v less often*	964	0.77	0.55	1.07	.125
Not stated *v all other categories*	189	1.11	0.73	1.70	.624
Frequency of close friends drinking					<.001
Never	72	Ref			
Less than monthly *v never*	188	0.63	0.30	1.34	.230
Monthly or fortnightly *v less often*	461	1.83	1.19	2.83	.006
At least weekly *v less often*	670	2.97	2.14	4.12	<.001
Not stated *v all other categories*	200	0.62	0.39	0.98	.040
Parents’ views					
Neutral/unacceptable	475	Ref			
Drinking acceptable	1,116	1.12	0.82	1.53	.466
Peer views					
Neutral/unacceptable	154	Ref			
Drinking acceptable	1,437	1.32	0.81	2.15	.269
Age of first drink					<.001
Age 13 or under	474	Ref			
Age 14 to 15 *(v 13 or under)*	533	0.90	0.65	1.25	.533
Age 16 or over *(v younger)*	411	0.27	0.20	0.37	<.001
Not stated	173	0.93	0.62	1.39	.719
Awareness of wider alcohol marketing activity					.032
Low awareness	183	Ref			
Medium *v low*	271	1.82	1.15	2.88	.011
High *v medium & low*	327	1.05	0.72	1.52	.795
Not stated *v all other categories*	810	0.86	0.67	1.10	.231
Own alcohol branded merchandise					
No/not sure	1,139	Ref			
Yes	452	1.45	1.10	1.91	.009
Number of social media apps used at least weekly					.029
Three or fewer	375	Ref			
Four or five	602	1.42	1.02	2.00	.040
Six more	614	1.59	1.12	2.24	.009
Participation with marketing on social media					.011
None	1,228	Ref			
One	204	0.91	0.62	1.33	.638
Two or more (max 5)	159	1.96	1.23	3.12	.005
Participation with user-created promotion					
No	1,187	Ref			
Yes	404	3.46	2.56	4.68	<.001

Base: Current drinkers (*n* = 1,591). Cases excluded due to missing values on one or more independent variables = 24.

Dependent variable: Higher-risk consumption (≥5 on AUDIT-C) in current drinkers (Higher-risk *n* = 696; Lower-risk *n* = 895).

Test of model coefficients: χ^2^ (34) = 573.81, *p* < .001. Nagelkerke R^2^=0.41.

Hosmer & Lemeshow χ^2^ (8) = 13.43, *p* = .098.

Cases correctly classified: 75%.

^a^Adjusted for all other variables in the model, Adj OR, adjusted odds ratio; Ref, reference category; 95% CI, 95% confidence interval.

### Association between participation with marketing and user-created promotion on social media and Brand identification

Participation with alcohol marketing on social media was not associated with brand identification (*β* = 0.01, *p* = 0.42) (Table 5). However, having medium (*β* = 0.08, *p* < .001), high (*β* = 0.08, *p* < .001), and not stated awareness of wider alcohol marketing activity (*β* = 0.09, *p* < .001) (vs. low awareness); ownership of branded merchandise (*β* = 0.09, *p* < .001); using a greater number of social media apps at least weekly (*β* = 0.10, *p* < .001); and participating with user-created promotion (*β* = 0.05, *p* < .001) were all positively associated with brand identification. Drinking behaviours were also significant factors. Compared with lower-risk drinkers, being a non-drinker was negatively associated with brand identification (*β*=-0.24, *p* < .001) while being a higher-risk drinker was positively associated with brand identification (*β* = 0.12, *p* < .001). Having a father who drinks at least weekly vs. less often or never (*β* = 0.06, *p* < .001); having close friends who drink at least weekly vs. less often or never (*β* = 0.04, *p* < .01); and perceiving that people my age consider drinking acceptable (*β* = 0.05, *p* < .01) were associated with greater brand identification. Demographic variables associated with greater brand identification included being older (*β* = 0.20, *p* < .001); white British (*β* = 0.09, *p* < .001); and in employment (*β* = 0.04, *p* < .01). Being from a more affluent IMD quintile (*β* = −0.03, *p* < .05) and male (*β* = −0.03, *p* < .05) was associated with lower brand identification.

**Table 5.** Association between participation with alcohol marketing and user-created promotion on social media and brand identification in young people.

	Unstandardized coefficients		Standardised coefficients		
Variables and reference categories	*B*	SE	*β*	*t*	*p*
Constant	−0.91	0.33		−2.79	0.005
Age	0.16	0.02	0.20	9.75	<0.001
Gender					
Male (*v female*)	−0.13	0.05	−0.03	−2.37	0.018
Ethnicity					
White British *(v other)*	0.50	0.07	0.09	7.12	<0.001
IMD Quintile					
(1: most deprived to 5: most affluent)	−0.04	0.02	−0.03	−2.18	0.029
Country					
Scotland *(v England)*	−0.15	0.08	−0.02	−1.80	0.072
Wales & Northern Ireland *(v Eng)*	−0.07	0.09	−0.01	−0.83	0.405
Educational status					
In education (*v not*)	0.03	0.14	0.003	0.22	0.830
Working status					
Working (*v not*)	0.34	0.12	0.04	2.92	0.004
Living status					
Living independently (*v with parents/adult family*)	0.11	0.10	0.01	1.06	0.288
Not stated (*v with parents or adult family*)	−0.11	0.31	−0.01	−0.37	0.711
Higher-risk drinking					
Non-drinker (*v lower risk current drinker*)	−1.03	0.09	−0.24	−11.45	<0.001
Higher-risk current drinker (*v lower risk current drinker*)	0.63	0.09	.122	7.29	<0.001
Frequency of mother drinking					
At least weekly (*v less often/never*)	−0.02	0.06	−0.01	−0.35	0.728
Not stated (*v less than weekly/never*)	−0.10	0.15	−0.01	−0.67	0.502
Frequency of father drinking					
At least weekly (*v less often/never*)	0.26	0.07	0.06	3.82	<0.001
Not stated (*v less than weekly/never*)	0.22	0.10	0.03	2.27	0.023
Frequency of close friends drinking					
At least weekly (*v less often/never*)	0.20	0.08	0.04	2.63	0.009
Not stated (*v less than weekly/never*)	−0.12	0.08	−0.02	−1.60	0.110
Parents’ views					
Drinking acceptable (*v neutral/unacceptable*)	0.06	0.07	0.01	0.87	0.382
Peer views					
Drinking acceptable (*v neutral/unacceptable*)	0.21	0.07	0.05	2.97	0.003
Age of first drink					
Age 13 or under *(v 14 to 15 years)*	0.06	0.09	0.01	0.68	0.496
Age 16 or over *(v 14 to 15 years)*	−0.10	0.10	−0.02	−0.99	0.320
Not stated *(v 14 to 15 years)*	−0.17	0.13	−0.02	−1.37	0.172
Awareness of wider alcohol marketing activity					
Medium (*v low awareness*)	0.52	0.10	0.08	5.07	<0.001
High (*v low awareness*)	0.50	0.11	0.08	4.74	<0.001
Not stated (*v low awareness*)	0.37	0.08	0.09	4.72	<0.001
Own alcohol branded merchandise					
Yes (*v no/not sure*)	0.52	0.08	0.09	6.93	<0.001
Number of social media apps use at least weekly	0.10	0.02	0.10	6.58	<0.001
Participation with alcohol marketing on social media	0.04	0.05	0.01	0.81	0.419
Participation with user-created promotion					
Yes (*v no/not sure*)	0.34	0.09	0.05	3.66	<0.001

Base: all participants, *n* = 3,298. Cases excluded due to missing data on one or more variables = 101.

Dependent variable: Number of (masked) brands correctly identified (0–8) (*M* = 2.63, *SD* = 2.12).

Model shown is final block, *F* (30, 3267) = 110.93, *p* < .001.

Final step model change: *F* (1, 3267) = 13.39, *p* < .001.

Total variance explained (Adj. *R*^2^ = 0.50). Durbin Watson = 1.999.

## Discussion

The results show that social media provides varied and dynamic ways for young people in the UK to participate with messages promoting alcohol, and that such participation is associated with higher-risk drinking. We extend understanding by showing participation in a demographically representative sample of young people in the UK, examining commercial and user-led content simultaneously, and highlighting that user-created promotion may also contribute to marketing goals by being associated with increased brand identification.

Around one-in-ten respondents had participated with at least one form of alcohol marketing on social media, including those under the legal purchase age. These findings are consistent with suggestions that newly legal drinkers are an important target audience for alcohol marketers (Hastings et al. 2010) and that age verification processes are only partially effective (Brooks 2010; Jones et al. 2014; Winpenny et al. 2014; Barry et al. 2015; Barry et al. 2016). The findings are also consistent with suggestions that audience participation is highly valued by marketers who, in turn, invest significant resources towards encouraging interactions with consumers (Nicholls 2012; Atkinson et al. 2014; Carah 2014; Moraes et al. 2014). Although our estimate of participation is lower than reported in recent research in the UK, these studies only sampled young adults above the legal purchasing age who are legitimate marketing targets (Critchlow et al. 2016). It is plausible that these conservative estimates are more representative of participation across adolescence. Research has also indicated that marketing on social media can be submerged below conscious awareness, which can create difficulties in recognising marketing intentions (Lyons et al. 2014). This may have led to under-reporting, particularly in younger adolescents less familiar with marketing. Nevertheless, participation with at least one form of alcohol marketing on social media by those under the legal purchasing age is still higher than reported in earlier UK research (Gordon et al. 2011), thus suggesting that participation with marketing on social media has increased.

Participation with two or more forms of marketing on social media was associated with higher-risk drinking, a finding consistent with previous research (Critchlow et al. 2016; de Bruijn et al. 2016; Lobstein et al. 2017; Buchanan et al. 2018). Although there was no association between participation with alcohol marketing on social media and brand identification, there was an association for awareness of wider alcohol marketing activity and ownership of branded merchandise. This reinforces the continued importance of conventional techniques in the ‘marketing mix’ and how different marketing strategies may influence consumers in varied ways (AFS 2017).

Approximately one-in-ten respondents had uploaded statuses or photos of themselves or their peers with alcoholic drinks and, consistent with previous research, this was associated with higher-risk consumption. Although the reported participation is lower than previously estimated in the UK, earlier research only focussed on those above the legal drinking age (Moss et al. 2015; Critchlow et al. 2017). As some drinking experience is a logical prerequisite for such content, it is possible that our conservative estimate is influenced by the varied drinking prevalence across adolescence ([PHE] Public Health England 2016). Indeed, the results show that participation with user-created promotion was higher in current drinkers and those above the legal purchasing age, and it is plausible that higher-risk drinkers are more likely to create social media posts about drinking and have peers who may also drink heavily. Nevertheless, participation with user-created promotion was double that of any form of alcohol marketing on social media. Possible reasons for this include peer content not being subject to age restrictions, that young people consider peer content more credible and authentic, that young people believe they are not the intended targets for marketing, or that interacting with peer content is more normalised than with marketing. That the association between participation with user-created promotion and higher-risk drinking was stronger than for commercial marketing may reflect that the former is not subject to regulations on appropriate content, can feature more explicit messages about intoxication, and is not required to promote lower-risk consumption. It is also possible that young people find peer messages more credible or congruent to their drinking identity than marketing and therefore more influential, although this is beyond the scope of this study. That user-created promotion was also associated with increased brand identification supports that such content may provide de facto free marketing for brands, even when not directly solicited. This reaffirms the utility of user-created content and why the alcohol industry considers it important (Nicholls 2012; Carah 2014).

There was also an association between social media apps used at least weekly and higher-risk drinking and brand identification. As the forms of digital marketing that we measured participation for was not exhaustive, it is plausible that social media use acted as a proxy for exposure to unmeasured forms of digital alcohol marketing, for example through music-streaming services (Ghosh 2015), display adverts or pop-ups (Critchlow et al. 2016), on-demand television (Siegel et al. 2016), and brand websites (Gordon 2011). This measure may have also acted as a proxy for content that is hard to recognise as marketing, such as sponsored video-blogs or articles on ‘pop culture’ websites (Stream Daily 2014; Captain Morgan 2016). Social media use may have also provide a proxy for awareness of, and participation with, unmeasured forms of user-created promotion, such as YouTube videos (Primack et al. 2015), smartphone apps (Weaver et al. 2014), and online drinking games (Wombacher et al. 2017).

To date, it is alcohol companies, media operators, and advertising bodies who have responded to commercial marketing on social media through self-and-co-regulatory frameworks (Portman Group 2009; IARD 2016; Facebook 2017; CAP 2018). This, however, comes against a backdrop of questions about the efficacy of such approaches, including research into digital marketing (Caroll and Donovan 2002; Brooks 2010; Carah et al. 2015; Noel and Babor 2017). There are only limited examples of statutory legislations being adapted to include social media marketing. France extended their Loi Evin law to cover intrusive online advertising (e.g. pop-ups and banners) and content which may appeal to young people (Cecchini and Belloni 2015; Gallopel-Morvan et al. 2017). From 2015, Finland also prohibited encouraging sharing of content on social media, online competitions, viral marketing, and ‘advergaming’ across new media devices (e.g. smartphones, tablets, and games consoles) (Montonen and Touminen 2017).

Establishing best practice for regulating alcohol marketing and user-created promotion on social media is complicated by several challenges (Brodmerkel and Carah 2013). For example, some marketing content is global, and this extraterritorial nature means it does not fit rigidly within the regulations of one country. This is further complicated by variations in marketing regulation between countries (WHO 2014) or international legislation adopting a ‘country of origin’ approach (IAS 2016). In Finland, it is acknowledged the legislation can only be enforced if the marketing is targeted at individuals in Finland, but not if someone accessed content from another country (Montonen and Touminen 2017). Establishing best practice is also complicated by continued new media innovation and the challenges posed by user behaviour, particularly user-created promotion and user-generated branding which does not always feature in a brand-controlled space (e.g. fan pages and video-blogs) (Portman Group 2009) and is not subject the same regulation of content or promotion of lower-risk consumption. For example, France’s initial revision to the Loi Evin only covered intrusive advertising (e.g. pop-ups), but not non-intrusive social media pages, micro-blogging accounts, online movies, or smartphone apps (Gallopel-Morvan et al. 2015). Further research is required to enhance understanding of how to reduce young people’s participation with messages promoting alcohol consumption on social media. Media and website operators, in particular, have an important role to play because of their expertise in media design, because their website terms and conditions provide a legitimate opportunity to address extra-territorial and user-created promotion, and because social media offers a valuable opportunity for lower-risk drinking messages or campaigns to be effectively targeted at young people.

This study does have limitations. The cross-sectional design does not demonstrate causality between participation with marketing or user-created promotion and higher-risk consumption, albeit the results do suggest either an initiating or reinforcing role (both of which are important) and longitudinal research supports a causal relationship (Boyle et al. 2016; McClure et al. 2016). We also only measured participation with marketing and user-created promotion. By not also measuring awareness, that is seeing content without actually participating, the findings likely under-estimate the association between social media and consumption. The retrospective self-report measure may also have led to under-reporting for participation, particularly for content where the marketing intentions were not explicitly clear (Lyons et al. 2014). We did not provide a ‘Not sure’ option for reporting participation, and this could have helped determine to what extent participants could not be sure whether they had participated with marketing but did not feel comfortable rejecting they had not. We also used apps used in the past week as a proxy for overall social media use and the data do not account for frequency or amount of time spent on each app, nor variability by demographic groups. It is possible that greater use of some apps may be more conducive to participation with commercial marketing and user-created promotion than others.

## Conclusion

The study represents, to our knowledge, the first survey of participation with both alcohol marketing and user-created promotion on social media in a representative sample of young people above and below the legal drinking age in the UK. The results show that young people’s use of social media provides varied opportunities to participate with alcohol-related content which, in turn, is associated with higher-risk consumption. That participation with user-created promotion was also associated with increased brand identification further highlights that social media may contribute to wider marketing goals, and not just increased consumption. Further research is required to explore awareness of, and participation with, a broader range digital marketing and user-created promotion in young people, and to engage stakeholders to identify the facilitators and barriers to mitigating the link between such content and higher-risk consumption.

## Supplementary Material

Supplemental Material
