# Supplementary material for: Participation with alcohol marketing and user-created promotion on social media, and the association with higher-risk alcohol consumption and brand identification among adolescents in the UK
Source: Addict Res Theory. 2019 Feb 19;27(6):515–26. doi: 10.1080/16066359.2019.1567715 (PMC6636897; doi:10.1080/16066359.2019.1567715)
Supplement: Supplemental Material [file IART_A_1567715_SM8873.docx]

| **Supplementary table 1:** Alcohol marketing channels included in measure of awareness | |
| --- | --- |
| 1. | Adverts for alcohol in newspapers or magazines |
| 2. | Adverts for alcohol on television |
| 3. | Adverts for alcohol on billboards |
| 4. | Adverts for alcohol on radio |
| 5. | Adverts for alcohol on YouTube, Tumblr, Facebook, Snapchat, Instagram or other social media |
| 6. | Famous people in films, music videos, on TV, or pictures in magazines with alcohol |
| 7. | Sport sponsorship |
| 8. | Special offers |
| 9. | Competitions |
| **Notes:**  Each scored Every – Not in the past month; Not sure. | |
